# Immunotherapy in Patients with Advanced Non-Small Cell Lung Cancer Lacking Driver Mutations and Future Perspectives

**DOI:** 10.3390/cancers14010122

**Published:** 2021-12-28

**Authors:** Ramon Andrade Bezerra De Mello, Rafael Voscaboinik, João Vittor Pires Luciano, Rafaela Vilela Cremonese, Giovanna Araujo Amaral, Pedro Castelo-Branco, Georgios Antoniou

**Affiliations:** 1 Division of Medical Oncology, Escola Paulista de Medicina, Hospital São Paulo, Universidade Federal de São Paulo (UNIFESP), Sao Paulo 04023-900, Brazil; med.voska@yahoo.com.br (R.V.); gioaraujoamaral@gmail.com (G.A.A.); 2 Faculdade de Medicina, Universidade Nove de Julho, Sao Paulo 04023-900, Brazil; joao.pires@uni9.edu.br (J.V.P.L.); rafaelacremonese@uni9.edu.br (R.V.C.); 3 Centro Médico Especialidades, Division of Medical Oncology, Hospital 9 de Julho, Sao Paulo 01409-002, Brazil; 4 Faculty of Medicine and Biomedical Sciences (FMCB), Campus de Gambelas, University of Algarve, 8003-139 Faro, Portugal; pjbranco@ualg.pt (P.C.-B.); dr.antoniou@gmail.com (G.A.)

**Keywords:** immunotherapy, advanced lung cancer, durvalumab, pembrolizumab, atezolizumab, ipilimumab, nivolumab, cemiplimab, PD1, PDL1, anti PD1, anti PDL-1

## Abstract

**Simple Summary:**

The following paper was developed as a reference for healthcare workers who are looking for information on advanced non-small cell lung cancers that lack driver mutations and their treatment with immunotherapy. The aim of the present study is to provide a reliable data source based on a review of the most current papers on the PD-1/PD-L1 and CTLA-4 inhibitors, as well as their use in medical practice.

**Abstract:**

From a complete literature review, we were able to present in this paper what is most current in the treatment with immunotherapy for advanced non-small cell lung cancer (NSCLC). Especially the use of immunotherapy, particularly inhibitors of PD-1 (programmed cell death protein 1), PDL-1 (programmed cell death protein ligand 1), and CTLA-4 (cytotoxic T-lymphocyte antigen 4). Since 2015, these drugs have transformed the treatment of advanced NSCLC lacking driver mutations, evolving from second-line therapy to first-line, with excellent results. The arrival of new checkpoint inhibitors such as cemiplimab and the use of checkpoint inhibitors earlier in the therapy of advanced and metastatic cancers has been making the future prospects for treating NSCLC lacking driver mutations more favorable and optimistic. In addition, for those patients who have low PDL-1 positivity tumors, the combination of cytotoxic chemotherapy, VEGF inhibitor, and immunotherapy have shown an important improvement in global survival and progression free survival regardless the PDL-1 status. We also explored the effectiveness of adding radiotherapy to immunotherapy and the most current results about this combination. One concern that cannot be overlooked is the safety profile of immune checkpoint inhibitors (ICI) and the most common toxicities are described throughout this paper as well as tumor resistance to ICI.

## 1. Introduction

Lung cancers are histologically divided into Small Cell Lung Cancer (SCLC), which is found in about 15% of patients, and Non-Small Cell Lung Cancer (NSCLC), which is the most common type, found in the remaining 85% of patients [1].

The incidence of non-small cell lung cancer in the year 2020 was approximately 2,206,771 new cases, while its mortality in this same year was 1,796,144 people, and this is, therefore, the cancer with the highest incidence and mortality in both sexes worldwide [2].

Major risk factors for NSCLC include cigarette smoking (even passive smoking can increase the risk of NSCLC by 3.6 times), exposure to urban air (11% of all NSCLCs in Europe are believed to be attributed to carbon-polluted urban air), and exposure to silica and asbestos (common in occupations such as mining). The most common histologic type of NSCLC is the adenocarcinoma [3,4].

Cancer is a multifactorial disease that develops due to the accumulation of genetic and epigenetic changes. Cancer cells as a whole share malignant patterns. Ten distinct biological characteristics, called Hallmarks of Cancer, acquired in the course of tumor development and can significantly determine the process of carcinogenesis. These Hallmarks are: capacity for proliferative signaling, inhibition of growth suppressor signals, evasion of immune destruction, mechanisms of resistance to apoptosis, promotion of inflammation in the tumor microenvironment, replicative immortality, genomic instability, induction of angiogenesis, ability to metastasize, and dysregulation of cellular metabolism. The acquisition of these functions in the course of tumorigenesis is possible with the appearance of genomic instability in tumor cells, which generates a propensity for mutations, such as chromosomal rearrangements [5].

The carcinogenesis of NSCLC is correlated with several of these driver mutations, such as the mutations in the epidermal growth factor receptor (EGFR) gene [6]. The tyrosine kinase activity of EGFR initiates signaling pathways related to cell proliferation and invasive growth (such as STAT3 and STAT5) [7]. Today we know that driver mutations are of utmost importance for programming individualized and standardized therapy of advanced non-small cell lung cancer of non-squamous etiology, and from these driver mutations we can choose the best treatment for our patient [8,9].

However, the incidence of driver mutations is low, as they affect only a portion of patients with non-small cell lung cancer of non-squamous etiology. Although EGFR is the most common mutation, it is found in only 15% of individuals (but the incidence can be as high as 43% in the subpopulation of Caucasian never-smoker patients) [10]. Followed by the ALK gene mutation, with approximately 5% of patients affected [11]. Other mutations, such as ROS1 and RET, have approximately a 1% involvement in the population [12].

Therefore, among the total incidence of non-small cell lung cancer cases, we still have a considerable number of patients who will not present any driver mutation and these patients should be treated with alternative therapies.

In addition, patients eventually develop resistance to first and second generation anti-EGFR treatments, with the main pathway leading to this resistance being a T790M mutation in the EGFR gene found in 62% of patients resistant to anti-EGFR therapy [13,14]. There are other mechanisms of resistance to therapies that combat driver mutations, such as additional point mutations in EGFR (C797S, C481S, G796D, among others), overexpression of cyclooxygenase-2 (COX-2), overexpression of IGFR-1, and amplifications of EGFR, HER2, and MET [15].

Based on this principle, we have the option of using immunotherapy for NSCLC that lack the most common driver mutations and also for those that become resistant to targeted therapy of driver mutations [16,17].

This therapy plays a key role in the treatment of patients with non-small cell lung cancer of squamous and non-squamous etiology who do not have driver gene mutations [18].

Inhibitors of immune checkpoints have become one of the most promising approaches in the field of cancer immunotherapy, particularly inhibitors of PD-1 (programmed cell death protein 1) and PD-L1 (programmed cell death protein ligand 1). PD-1 is a cell surface immunoglobulin, expressed on T and pro-B cells, that binds to its receptor PDL1, causing the death of lymphocytes and thereby suppressing the immune response. Monoclonal antibodies that bind to the PD-1 receptor block its interaction with ligands [19,20,21,22].

Blocking the PD-1/PD-L1 signaling pathway allows activated T cells to secrete cytokines to restore the antitumor immune response in the setting of treating advanced NSCLC without driver mutations.

PD-1 or PD-L1 checkpoint inhibitors have routinely become part of the clinical approach for this type of cancer, which justifies the importance of researching PD-1/PD-L1 status in patients with advanced non-small cell lung cancer [23]. In addition to the PDL pathway, cancer cells can also inhibit the activation of lymphocytes and lead them to apoptosis by expressing molecules that bind to CTLA-4 (cytotoxic T-lymphocyte antigen 4), an inhibitory receptor present on the surface of lymphocytes [24,25].

## 2. Immune Checkpoint Inhibitors as Second-Line Therapy for Advanced NSCLC

The PD-1/PD-L1 blockade therapies significantly improve the response rate, prolong patient survival, and have limited adverse effects either in monotherapy or in combination therapy for advanced NSCLC.

Several studies have proven their efficacy in patients who have failed first-line treatment [26]. Nivolumab (Opdivo^®^), a PD-L1 inhibitor, was approved by the Food and Drug Administration (FDA) [27], when studies demonstrated that Nivolumab, associated with a CTLA-4 blocker (Ipilimumab) or in monotherapy, generated increased overall survival when compared to docetaxel in patients with metastatic NSCLC of squamous etiology who had already failed to respond to platinum-based systemic treatment [28,29,30].

The clinical trial Checkmate 057 evaluated the role of Nivolumab in patients with non-squamous NSCLC who had disease progression even after intervention with platinum-based chemotherapy. Nivolumab was administered at a dose of 3 mg/kg every 2 weeks, and the control group received docetaxel at a dose of 75 mg/m^2^ every 3 weeks. Patients who received Nivolumab achieved a higher overall survival rate at 18 months of follow-up (39%) compared with docetaxel (23%), regardless of levels of PD-L1 expression [31].

Very similarly, the Checkmate 017 trial evaluated patients with staged III and IV squamous-cell NSCLC who had recurrence after a platinum-based chemotherapy. Patients were randomized to receive either Nivolumab or Docetaxel, the primary endpoint was overall survival and among the secondary end points, it is worth mentioning efficacy according to tumor PD-L1 expression. The median overall survival was 9.2 months with Nivolumab and 6 month with Docetaxel (HR 0.59; *p* < 0.001) and the positivity of PDL-1 expression did not impact the trial result, it was not beneficial neither in terms of prognostic nor in the predictive of a better response to the treatment [32].

Pembrolizumab (Keytruda^®^) is the most widely used checkpoint inhibitor and has received FDA approval in the US for the treatment of advanced NSCLC [33,34]. It has been proven effective by the KEYNOTE 010 trial. This double-blind phase 3 study has compared Pembrolizumab at two different doses (10 and 2 mg/kg) against Docetaxel, the standard of care chemotherapy regimen at the time. Pembrolizumab significantly improved overall survival at both doses when compared with chemotherapy, at the 2 mg/kg dose the hazard ratio (HR) was 0.71 (95%CI 0. 58–0.88 *p* = 0.0008) and at 10 mg/kg the HR was 0.61 (95%CI 0.49–0.75 *p* < 0.0001). Both doses were shown to be well tolerated and with fewer Grade 3–5 side effects than Docetaxel [34]. Studies show that Pembrolizumab demonstrated overall survival gain especially in patients who had the expression test for PDL-1 with positive rate greater than 1%, and the highest OS gains were achieved for those patients who had PDL-1 status greater than 50% [35,36].

Another checkpoint inhibitor is Atezolizumab (Tecentriq^®^), approved by the FDA. It has a different mechanism of action than the other inhibitors—this monoclonal antibody binds directly to PD-L1 and promotes a double blockade of PD-1 and B7 receptors [37]. Atezolizumab was popularized by the OAK trial. The outcome of the study was satisfactory, with survival gain even in patients who lacked PDL-1 detection, which points out that Atezolizumab is a good option for second-line therapy, i.e., for advanced NSCLC in patients who have already received platinum-based therapy [38].

However, in the OAK trial, the stratification of PDL-1 positivity was different from the studies cited so far. Patients were stratified as IC1/2/3 or TC1/2/3 for those with PDL-1 expression of 1% or more in tumor cells, TC2/3 or IC2/3 for those with PDL-1 positivity of 5% or more in tumor cells and TC 3 for those with 50% or more PDL-1 positivity in tumor cells and in a complementary way IC3 for those with 10% or more positivity and finally IC0 or TC 0 were for those with less than 1% of PDL-1 positivity in tumor cells.

This was due to a score previously used in the POPLAR phase II work where the positivity of PDL-1 to the immunohistochemical marker VENTANA SP 142 and were categorized according to the total percentage of PDL-1 expression in the studied tumor area [39].

In 2021, Cemiplimab (Libtayo^®^) had its approval by the FDA [40]. Studies on the efficacy of this drug are still incipient. The phase 1 cohort by Moreno et al. tested Cemiplimab in patients with advanced NSCLC (stage III/IV) and refractory to conventional therapy. Cemiplimab was administered in doses of 200 mg every 2 weeks intravenously. Five had a partial response to the drug, four had stable disease, and 10 had progressive disease. The objective response rate was 25.0% (95% CI: 8.7–49.1%) [41].

## 3. Immune Checkpoint Inhibitors as First-Line Therapy for Advanced NSCLC

Patients with advanced non-small cell lung cancer undergo palliative treatment with the goal of increasing their survival as well as providing greater quality of life. The choice of treatment depends on histology, the level of PD-L1 expression and/or the presence or absence of contraindications [42]. According to the National Comprehensive Cancer Network (NCCN) Guidelines, Pembrolizumab monotherapy can be used as a first-line therapy for non-squamous NSCLC with high PD-L1 expression (≥50 percent expression), as seen in the Keynote 024 and Keynote 042 trials.

Both papers evaluated the first-line use of Pembrolizumab in comparison with standard chemotherapy. In Keynote 024, the selected patients had a rate of PDL-1 expression of at least 50%, and the main outcome was gains in progression free survival (PFS). Patients who received Pembrolizumab had a higher PFS, showing a HR of 0.5 (95% CI 0.37–0.68 *p* < 0.001), as well as a higher OS, showing a HR of 0.6 (95% CI 0.41–0.89 *p* < 0.005).

The Keynote 042 trial recruited NSCLC patients with PDL-1 expression higher than 50%, but patients with PDL-1 expression >20% and >1% were also analyzed. There was a gain in OS in all groups tested in comparison with standard chemotherapy. For those with PDL-1 expression >50%, the HR was 0.69 (95% CI 0.56–0.85, *p* = 0.0003), for those with PDL-1 expression ≥20%, the HR was 0.77 (95% CI 0.64–0.92, *p* = 0.0020), and for those with PDL-1 expression ≥1%, the HR was 0.81 (95% CI 0.71–0.93, *p* = 0.0018) [43,44].

However, in a post-analysis of KEYNOTE 042, pembrolizumab monotherapy failed to demonstrate an overall survival benefit in patients with PDL-1 of 1 to 49%. Even so, Pembrolizumab has shown to be better for these patients than standard chemotherapy, with better tolerability and therefore being an option for patients who decline traditional chemotherapy or who are unfit to receive standard chemotherapy [45].

The newer drug cemiplimab was tested in the EMPOWER Lung 1 trial, and demonstrated gains in OS. By the end of the study, a survival HR of 0.57 (95%CI 0.42–0.77 *p* = 0.002) was achieved in patients with a PDL-1 expression >50% [46].

Moving away from the PD-1/PD-L1 axis. There are other FDA-approved checkpoint inhibitors, such as: ipilimumab (Yervoy^®^) in advanced NSCLC, which has as its mechanism of action the blockade of CTLA-4, which prevents the negative regulation of T-lymphocyte activation, leading to potentiation of lymphocyte activation and, consequently, decreased tolerance to cancer antigens [47]. Ipilimumab was associated with Nivolumab in a phase 3 study by Hellmann et al. This drug combination generated an overall survival of 17.1 months (95% CI, 12.7 to 16.7) among patients with a PD-L1 expression level of 1% or more, whereas chemotherapy generated an overall survival of 14.9 months (*p* = 0.007). The overall survival rates at the end of 2 years were 40.0% for iplimumab + nivolumab and 32.8% for chemotherapy [48].

Along with the first-line treatment decision, the patient should be advised to cease tobacco use [49]. However, if there is difficulty in obtaining the indicated therapies preferentially exposed as first-line, there is the possibility of cytotoxic chemotherapies recommended in the NCCN to replace them, but not with the same efficacy and toxicity patterns.

## 4. The Use of Combined Therapy in Advanced and Metastatic Non-Small Cell Lung Cancer

### 4.1. Combined Immunotherapy plus Chemotherapy Treatment for First-Line Metastatic NSCLC

Recently, several trials have studied the combination of immunotherapy with chemotherapy for patients with metastatic tumors who have never undergone any prior therapy. These studies show positive results regardless of PDL-1 status, being a good option for patients who have low PDL-1 positivity

The Keynote 189 study evaluated the use of Pembrolizumab plus platinum-based chemotherapy plus pemetrexed in patients with non-squamous NSCLC. Patients received pembrolizumab plus platinum-based chemotherapy plus pemetrexed for four cycles followed by maintenance pembrolizumab for up to 35 cycles versus platinum-based chemotherapy plus pemetrexed plus placebo followed by placebo maintenance. The study demonstrated that in treatment-naive metastatic NSCLC cancer patients, there was a significant gain in overall survival with the association of pembrolizumab with chemotherapy, and this result was seen in all categories of PDL-1 expression positivity. OS in 12 months of 69.2% in the pembrolizumab group vs. 49.4% in the placebo group (HR 0.49; *p* < 0.001) reaching HR 0.59 in patients with PDL-1 expression < 1% and HR 0.55 in patients with between 1 and 49% expression, Hazard Ratio for disease progression or death 0.49 [50].

Another study that demonstrated positive overall survival with the association of chemotherapy, anti VEGF and immunotherapy in metastatic NSCLC patients was the IMPOWER 150. Patients with metastatic or recurrent non-squamous NSCLC, who had never received chemotherapy, were randomized to receive Atezolizumab plus carboplatin plus paclitaxel versus atezolizumab plus carboplatin plus paclitaxel and bevacizumab versus the group receiving only the combination of carboplatin plus paclitaxel plus bevacizumab without Atezolizumab. After 4 to 6 cycles, patients continued to receive Atezolizumab, bevacizumab, or a combination of the two until disease progression or limiting toxicity.

Once again the combination of chemotherapy, immunotherapy and, in this trial, associated with anti-VEGF demonstrated gain in overall survival and progression-free survival independent of PDL-1 Status. With a PFS hazard ratio of 0.62 (*p* < 0.001) for the group that received the combination with Atezolizumab and an overall survivor hazard ratio of 0.78 (*p* = 0.02) [51].

The Keynote 407 trial compared the combination of immunotherapy and chemotherapy in patients with metastatic NSCLC of squamous etiology. Patients were randomized to receive carboplatin plus paclitaxel or nab-paclitaxel plus Pembrolizumab or placebo. Patients received four cycles of combination therapy followed by maintenance with Pembrolizumab or placebo for up to 35 cycles. The study resulted in a significantly longer overall survival for the group that received Pembrolizumab compared to the group that received placebo, the study was benefic regardless of PDL-1 expression. The hazard ratio for overall survival was 0.64 (*p* < 0.001) in favor of the group that received Pembrolizumab. Progression-free survival also benefited patients who used combination chemotherapy with Pembrolizumab at HR 0.56 (*p* < 0.001). Making it an option for those patients with metastatic disease of squamous etiology independent of PDL-1 status [52].

Lastly, the CHECKMATE 9LA study evaluated the combination of Nivolumab an anti PD-1 plus CTLA-4 inhibitor Ipilimumab in combination with platinum-based chemotherapy versus chemotherapy alone. This study involved patients with metastatic or relapsed NSCLC. Patients could have squamous or non-squamous histology and could have undergone previous neoadjuvant, adjuvant, or chemoradiotherapy therapy provided they had discontinued treatment at least 6 months prior to recruitment. Patients received Nivolumab plus Ipilimumab plus platinum-based chemotherapy depending on histology, for two cycles followed by Nivolumab + Ipilimumab maintenance until limiting toxicity. Platinum-based therapies were given according to histological type, for non-squamous carboplatin or cisplatin plus pemetrexed and for squamous carboplatin plus paclitaxel. Patients in the non-squamous histological group who received only platinum plus pemetrexed-based chemotherapy could do maintenance with pemetrexed. Once again, the study showed positive results of overall and progression-free survival for the group that received the combination of immunotherapy and chemotherapy. The combination was beneficial in all patients of the combined therapy group regardless of PDL-1 status. The overall survival hazard ratio was 0.69 (*p* = 0.00065) with a median survival of 15.6 months versus 10.9 months for the benefit of the combined immunotherapy group, with a progression-free survival of 6.8 months for the combination immunotherapy group versus 5 months in the chemotherapy alone group (HR 0.7; *p* = 0.00012) [53].

Therefore, for patients with PDL-1 status ≤ 5%, the use of immunotherapy combined with chemotherapy and/or VEGF inhibitors appears as an excellent treatment option, bringing beneficial results both for overall survival and for progression-free survival in addition to good tolerance and a good safety profile for patients.

### 4.2. Combined Therapies for Treatment of Advanced Non-Metastatic NCLC

Within advanced, non-metastatic, but inoperable lung cancer the anti-PDL1 agent Durvalumab has the ability to prevent disease progression when used as a consolidation treatment after chemotherapy associated with radiotherapy. Radiation can have both immunosuppressive effects and immune-modulatory effects. In terms of immune system suppression, radiotherapy can recruit myeloid cells that promote tumor growth, can cause dysfunction of T cells and can lead to the up-regulation of PD-L1 expression, inhibiting the anti-tumor response of T-cells. However, radiotherapy can also foster the activation of the immune system, because the death of tumor cells by radiation leads to the release of tumor-specific antigens, which increases dendritic-cell presentation and T cell activation, and therefore more immune-dependent cell death. Because of these conflicting effects of radiotherapy on the immune system, many clinical trials have sought to determine the clinical outcomes of combining radiation with immune checkpoint blockers [54].

The use of Durvalumab comes from the Pacific trial, which recruited patients with stage III unresectable NSCLC whose disease had not progressed after concurrent platinum-based chemotherapy and radiotherapy. The approval of this line of treatment was based on an interim analysis of Progression Free Survival of the patients of a randomized, double-blind, placebo-controlled trial conducted in 713 patients with unresectable stage III NSCLC. The patients received Durvalumab at a dose of 10 mg/kg every two weeks (N = 473) or placebo for up to 12 months (N = 236). The study pointed to benefits for sequential treatment with Durvalumab, with median progression-free survival of 16.8 months (95% CI, 13.0–18.1) compared with the PFS of 5.6 months of patients that received placebo (HR = 0.52, 95% CI, 0.42–0.65, *p* < 0.0001). This drug is a 100% humanized high-affinity monoclonal antibody (immunoglobulin G1 kappa) that selectively blocks the interaction of PD-L1 with PD-1 and CD80 (B7.1), while leaving the PD-1/PD-L1 interaction intact.

The most recent update from the Pacific trial has been published in May 2021, and shows a new median follow-up duration of 34.2 months (range: 0.2–74.7 months). The updated OS is of 47.5 months for durvalumab versus 29.1 months for placebo, and the 60-month OS rates were 42.9% with durvalumab and 33.4% with placebo, showing a continuous benefit of durvalumab-based treatment in advanced, unresectable NSCLC [55,56,57,58,59,60].

An interaction between immune response and radiotherapy has been experimentally proven, in which radiation-mediated leukocyte infiltration can be driven by changes in leukocyte extravasation, inducing infiltration of a range of leukocytes such as: regulatory T cells (Tregs), cells Effector cells, natural killer (NK) cells, CD11b + cells such as myeloid-derived suppressor cells (MDSCs), and macrophage-associated tumor cells (TAMs). Thus, radiotherapy alone is a double-edged sword in immunology, on the one hand it prevents tumor growth, on the other hand, radiotherapy increases infiltration by Treg and CD11b + cells, including MDSCs and TAMs, which are associated with a microenvironment tumor immunosuppressive and in cancer patients observed an unfavorable outcome. However, CD11b + mediated immunosuppression may be transient and later replaced by the influx of effector T cells. Furthermore, in combination of radiotherapy with immunotherapy, the accumulation of CD11b + cells can be avoided and immunostimulatory effects seem to prevail, which makes the combination of immunological checkpoint inhibitors-radiotherapy of clinical interest and is in full debate, to determine if possibly effective [61].

In a meta-analysis of immunological checkpoint inhibitors and radiotherapy in advanced NSCLC cancer, the effect of adding radiotherapy to immunotherapy on both 1-year and 3-year overall survival was observed across several studies, finding a statistically significant benefit across patients locally advanced and metastatic patients. In that same meta-analysis, the effect of the combination of immune checkpoint inhibitors-radiotherapy on progression-free survival at 1 year and at 3 years was also observed. It was noted that there was a statistically significant benefit in both the ICI-RT vs. RT alone, how much ICI-RT vs. ICI alone, an increase in progression-free survival was also observed in patients with advanced or metastatic NSCLC. In addition, that article reported the safety of combination therapies, showing maximum grade 3 or 4 adverse events of any cause occurred in 31.3% of patients in the ICI-RT group and in 25.9% of those in the single treatment. Furthermore, no statistically significant difference was found between the most frequent adverse events, that is, it is safe for the patient [62].

While many studies attempt to characterize new pathways of treatment parallel to PD-L1 blockers, others are still aiming to identify the impact of these ICI in susceptible populations. The ongoing IMpower study investigating the use of adjuvant Atezolizumab recently published new results in 2021. In a phase III study, named IMpower010, adjuvant Atezolizumab was tested against best supportive care in patients with stage IB-IIIA NSCLC who were previously treated with platinum-based chemotherapy. The chemotherapy consisted of a maximum of four cycles of 21 days each of cisplatin therapy, associated with pemetrexed, docetaxel, gemcitabine, or vinorelbine and Atezolizumab was administered in 16 cycles of 1200 mg each, every 3 weeks. After a median follow-up of 32.2 months, atezolizumab showed the most benefit in extending disease free survival for patients with PD-L1 expression in 1% or more of tumor cells (*p* = 0.0039) [63,64].

The association between radiotherapy and the specific ICI Pembrolizumab was tested by the PEMBRO-RT and MDACC phase 2 trials. The patients with metastatic NSCLC received either Pembrolizumab alone (200 mg every 3 weeks) or in combination with radiotherapy (24 Gy in three fractions in the PEMBRO-RT trial and 50 Gy in four fractions or 45 Gy in 15 fractions in the MDACC trial) in order to test the treatment association. The pooled analysis of both trials determined a response rate of 19.7% in the Pembrolizumab arm (15 of 76 patients) versus 41.7% in the Pembrolizumab plus radiotherapy arm (30 of 72 patients) (odds ratio 2.96, 95% CI 1.42–6.20; *p* = 0.0039). Besides showing a higher response rate, the patients who received association therapy also showed prolonged survival. The median progression-free survival was 4.4 months in the Pembrolizumab arm (ranging from 2.9 to 5.9 months), while the Pembrolizumab plus radiotherapy had a median of 9.0 months, ranging from 6.8 to 11.2 months (hazard ratio 0.67, 95% CI 0.45–0.99; *p* = 0.045). Finally, the median overall survival was 8.7 months (6.4 to 11.0 months range) in the Pembrolizumab arm against an OS of 19.2 months (14.6 to 23.8 months range) in the combination therapy arm (0.67, 0.54–0.84; *p* = 0.0004) [65].

## 5. Toxicities of Use of Checkpoint Inhibitors

One of the options for the first line of treatment for NSCLC is Pembrolizumab, either in monotherapy or in combined therapy. Pembrolizumab, like other checkpoint inhibitors, can cause adverse reactions that hinder the follow-up of the patients and treatment adhesion [65]. These reactions are immune system-related adverse effects (irAEs) that result from nonspecific activation of the immune system and induction of autoimmune tissue destruction or alteration, and they can affect the system as a whole. Most toxicities are discrete, but their intensity can vary between scenarios of adjuvant therapy and therapy for metastatic disease. In some studies, the reported incidence for irAEs of any grade due to checkpoint inhibitor monotherapy is approximately 90%. There is significant variation in the definitions of the severity of toxicity across guidelines, with variations in the signs and symptoms attributed to the same pathophysiology, thus making it difficult to obtain accurate incidence and accurate data on incidence and prevalence in clinical trials.

The irAEs can include dermatologic, gastrointestinal, hepatic, and endocrine inflammatory events. Dermatological symptoms can include maculopapular rash and pruritus. Amongst the gastrointestinal side effects, the most common symptom is diarrhea, but nausea and abdominal pain can also be common. Hepatic events include hepatitis, characterized by elevated levels of aspartate aminotransferase (AST) and alanine aminotransferase (ALT), with or without jaundice, caused by elevated bilirubin. In regards to endocrine toxicity, the effects of immunotherapy can be more prominent in the thyroid and pituitary glands, causing conditions such as hypothyroidism, hyperthyroidism, hypopituitarism, or hypophysitis. The most commonly observed pulmonary events were pneumonitis. The musculoskeletal and reumatologic events are represented by polyarthritis. In the renal system, the reported events are interstitial nephritis, granulomatous nephritis, lupus-like nephritis, and renal failure. The hematological events that can occur are hemolytic anemia, red blood cells aplasia, neutropenia, thrombocytopenia, myelodysplasia, and type A hemophilia. The diagnosis of cardiovascular reactions to immunotherapy are still a great challenge, and probably for this reason, studies report them to be quite rare.

The adverse effects to immunotherapy are mostly mild to moderate in severity. The severe, life-threatening reactions include, for example, severe colitis, pneumonitis, encephalitis, toxic epidermal necrolysis, myocarditis, and autoimmune type I diabetes mellitus (DM1) causing diabetic ketoacidosis [66,67].

The adverse effects are classified according to the degree of toxicity, with grade 1 being the mildest and grade 5 being toxicity-related death, defined by the Common Terminology Criteria for Adverse Events (CTC-AE). Most of these adverse events can be reversed with the use of oral or intravenous corticoids, but if grade 4 toxicity occurs, treatment must be stopped permanently, and the patient will demand a different method of individualized treatment [68,69].

Resistance to immunotherapy occurs when the patient does not respond to PD-L1 inhibition. This resistance can be primary or secondary. Primary resistance is due to the presence or absence of tumor-infiltrating lymphocytes (TILs) and the presence or absence of PD-L1 expression in the tumor microenvironment of lung cancer. Secondary or acquired resistance to tumor infiltrating immune cells occurs after a documented response to immunotherapy, associated with increased markers of adaptive immunity, T-cell activation and regulation [70].

## 6. Future Perspectives

One important concern of the current research in immunotherapy in NSCLC is how to overcome eventual resistance to immune checkpoint blockers. A search of the National Institute of Health’s clinical trials registry (clinicaltrials.gov) shows that there are 24 ongoing studies on immunotherapy resistance in advanced NSCLC. In this sense, as a future perspective, clinical trials have sought to better understand resistance in immunotherapies and the best form of treatments for patients who present tumor resistance to ICI.

A 2021 prospective study from Shanghai Chest Hospital in the stage of recruitment aims to characterize molecular markers of primary and secondary resistance to immunotherapy via DNA sequencing (through the techniques of whole exome sequencing and targeted sequencing). The study will include the analysis of DNA samples and fresh tumor tissue from 250 patients who receive anti-PD-1/PD-L1 monoclonal antibodies and still have active disease (according to the RECIST criteria). The study will also assess the objective response rate, progression-free survival and overall survival for PD-L1 blockers in the patients analyzed.

Another current study investigating PD-L1 blocker resistance comes from Sheba Medical Center in Israel. This study is a single-arm, phase 2a trial testing the safety and efficacy of treatment with Durvalumab, Tremelimumab, and radiotherapy in patients with advanced NSCLC who failed first-line immunotherapy with anti-PD1 or anti-PD-L1 antibodies. The progression of the disease must be documented on radiologic scans and on the RECIST scale. The study will assess the response rate of the new treatments on the immunotherapy-resistant patients, alongside incidence of adverse effects, progression-free survival and overall survival. The study also plans to collect blood, tumor, and microbiome samples to analyze the change in biomarkers in a time span of 5 years.

The clinical study called PIONEER, which is being carried out by Assistencia Publique Hopitaux De Marseille, is a phase II study that aims to assess the possibilities of overcoming resistance to ICIs or ICI monotherapies in combination with platinum-based chemotherapies, with precision experimental immunotherapies combined with Durvalumab in the second, third, or fourth line in patients with advanced NSCLC and disease progression after first line PD-1 or PD-L1 inhibitor. Afterwards, some supplementary blood and tissue samples will be collected in order to identify personalized biomarkers from the patients, correlating them with the efficacy outcomes, in order to better understand the resistance mechanisms and improve their future treatment. There is an estimated enrollment of 120 patients who will be allocated for treatment to be performed using the eCRF randomization module, dividing into four arms, the first being a Durvalumab + Monalizumab combination to target a PD-L1 co-inhibitory pathway, the second a Durvalumab + combination oleclumab, to address the limitations of anti-tumor T cell immunity caused by adenosine receptor signaling, the third a combination of Durvalumab + Ceralasertib, to potentiate anti-tumor T cell responses, and the fourth a standard maintenance third- or fourth-line chemotherapy (Docetaxel). The study is expected to end in February 2024.

## 7. Conclusions

In conclusion, the advancement of personalized therapy, as well as of immune therapy, offer an optimistic view to patients who lack driver mutations, since we increasingly understand the importance of regulating T cell immunity and its clinical application.

The importance of using checkpoint inhibitors in patients lacking driver mutations brings an additional treatment option with safe and tolerable side effects. Patients with advanced non-small cell carcinomas, in both histology, adenocarcinoma, and squamous, are favored with the use of checkpoint inhibitors in both non operable advanced and metastatic treatment lines.

In the second line of treatment, with studies dating from the year 2015, we can see how patients in both histology and regardless of the status of PD-L1 benefit from the use of checkpoint inhibitors with increased overall survival, progression-free survival and with toxic effects that are well tolerated by patients (Table 1).

More recently, checkpoint inhibitors have also shown to be effective in the first line of metastatic tumors, once again showing their efficiency in overall survival, progression-free survival and showing acceptable toxicities regardless of the histology of non-small lung cancer.

Expression of PD-L1 is important in defining therapy, with patients with positive, high-expression PD-L1 being able to benefit from checkpoint inhibitors, while those who express less are benefited by the combination of chemotherapy and immunotherapy (Table 2).

In patients with locally advanced disease, immunotherapy also proved to be extremely important since the use of Durvalumab as consolidation therapy increased progression-free survival, time to relapse, duration and response time, and decreased metastases as seen in the PACIFIC study.

The Radiotherapy associated with immunotherapy shown to be important in improving overall survival for both locally advanced and metastatic NSCLC, in addition to significantly influencing progression-free survival. Currently, the combination of immunotherapy with radiotherapy has been extensively studied due to a positive interaction between immunotherapy and radiotherapy and most likely, in a near future, be incorporated into the standard treatment hall of advanced and metastatic NSCLC lacking drivers’ mutations.

In any case, immunotherapy proved to be extremely satisfactory and with encouraging results for patients who do not have driver mutations. Thus, it appears as an option for these patients in any scenario of advanced inoperable disease to metastatic disease in the first or second line.

At the moment we are hoping that with the publication of the results of the new papers on checkpoint inhibitors we will have new and better treatment possibilities for patients with non-small cell lung cancer who lack driver mutations [71].

## Figures and Tables

**Table 1:** Second Line Immunotherapy approval for metastatic NSCLC.

Trial Name	Phase	Line of Treatment	Study Design	OS	HR (95%CI) & *p*
Checkmate 017	III	Second line	Nivolumab vs. Docetaxel	9.2 m with Nivolumab vs. 6 m with Docetaxel	0.62 (0.47 to 0.8) *p* < 0.0001
Checkmate 057	III	Second line	Nivolumab vs. Docetaxel	12.2 m with Nivolumabe vs. 9.4 m with Docetaxel	0.73 (0.59 to 0.8) *p* = 0.002
Keynote 010	III	First and Second line	Pembrolizumab 2 mg/kg vs. Pembrolizumab 10 mg/kg vs. Docetaxel	10.4 m with Pembrolizumab 2 mg/kg vs. 12.7 m with Pembrolizumab 10 mg/kg vs. 8.5 m with Docetaxel	0.71 (0.58 to 0.88) *p* = 0.0008 for Pembrolizumab 2 mg/kg and 0.61 (0.49 to 0.75) *p* < 0.0001 for Pembrolizumab 10 mg/kg
Oak Trial	III	Second line	Atezolizumab vs. Docetaxel	13.8 m with Atezolizumab vs. 9.6m With Docetaxel	0.73 (0.62 to 0.87) *p* = 0.0003

**Table 2:** Types of therapy according with PDL-1 Expression and histology.

PDL-1 Expression	Histological Type	Line of Treatment	Therapy Options
PDL-1 > 1% and ≤50%	Squamous	First Line	Platinum-based with paclitaxel or nabpaclitaxel combination plus immunotherapy Pembrolizumab or Nivolumab plus Ipilimumabe
PDL-1 > 1% and ≤50%	Non-squamous	First Line	Platinum-based chemotherapy with pemetrexede or anti VEGF plus immunotherapy with Pembrolizumab or Atezolizumabe or Nivolumab plus Ipilimumabe
PDL-1 > 50%	Squamous	First Line	Nivolumab plus Ipilimumab Pembrolizumab
PDL-1 > 50%	Non-squamous	First Line	Nivolumab Nivolumab plus Ipilimumab Pembrolizumab Atezolizumab Cemiplimab
